# Risk factors and outcomes associated with persistent vancomycin resistant Enterococcal Bacteremia

**DOI:** 10.1186/s12879-022-07864-8

**Published:** 2022-11-16

**Authors:** Emily Fox, David Ha, Mark Bounthavong, Lina Meng, Emily Mui, Marisa Holubar, Stanley Deresinski, William Alegria

**Affiliations:** 1 University of Texas MD Anderson Cancer Center, 1515 Holcombe Blvd, Houston, TX 77030 USA; 2 Stanford Antimicrobial Safety and Sustainability Program, Stanford, CA USA; 3 Division of Infectious Diseases and Geographic Medicine, Stanford University School of Medicine, Stanford, CA USA; 4 Department of Quality, Stanford Health Care, 300 Pasteur Drive, Stanford, CA 94305 USA; 5 UC San Diego Skaggs School of Pharmacy and Pharmaceutical Sciences, CA San Diego, USA

**Keywords:** Blood stream infections, Persistent bacteremia, Gram positive resistance, VRE, Enterococcus

## Abstract

**Background:**

Prior studies have identified that vancomycin resistant enterococcus (VRE) bacteremia that persists for four days or more is an independent predictor of mortality. Despite this, there is no published data to identify those patients at highest risk of developing persistent VRE bacteremia.

**Methods:**

This was a single center, retrospective, case-control study of adult patients with a VRE bloodstream infection (BSI). Case patients were those with persistent bacteremia (≥ 4 days despite VRE-directed therapy) and control patients were those with non-persistent bacteremia. Logistic regression was used to assess risk factors associated with persistent VRE BSIs. Secondary outcomes included in-hospital mortality, recurrent bacteremia, and breakthrough bacteremia.

**Results:**

During the study period, 24/108 (22%) patients had persistently positive blood cultures. Risk factors for persistent bacteremia included severe neutropenia (OR 2.13), 4 out of 4 positive index blood cultures (OR 11.29) and lack of source control (OR 11.88). In an unadjusted analysis, no statistically significant differences in in-hospital mortality (58% versus 40%; p = 0.121), recurrent bacteremia (17% versus 6%; p = 0.090), or breakthrough bacteremia (13% versus 7%; p = 0.402) were observed between groups.

**Conclusion:**

Patients with severe neutropenia, 4 out of 4 positive index blood culture bottles, and lack of source control were more likely to develop persistent VRE bacteremia despite directed antibiotic treatment.

**Supplementary Information:**

The online version contains supplementary material available at 10.1186/s12879-022-07864-8.

## Background

Enterococcal bloodstream infections (BSIs) are associated with significant mortality, with some estimates as high as 50% [1–6]. Vancomycin resistance has also been identified as an independent predictor of all-cause and infection-related mortality in patients with enterococcal BSIs [3, 4]. Although no consensus definition of “persistent” vancomycin resistant enterococcus (VRE) bacteremia exists, multiple studies have demonstrated that BSIs that take four days or more to clear are independently associated with mortality [7–11]. A recently published prospective observational study similarly reported that failure of VRE bacteremia to clear within 4 days of the index culture was the strongest predictor of poor outcomes [8]. While several studies have attempted to define persistent *Staphylococcus aureus* bacteremia and associated risk factors, similar studies are not available for VRE [12].

In addition to the lack of data to identify those at highest risk of developing persistent VRE bacteremia, optimal management remains controversial [13–16]. Enterococci express intrinsic and acquired resistance mechanisms that significantly limit the number of treatment options available. Clinical uncertainty is further exacerbated when VRE bacteremia persists despite directed therapy. Recognizing these challenges, both the Centers for Disease Control and Prevention and the World Health Organization have designated VRE as a high-priority multi-drug resistant organism that poses a threat to public health.

Given the association with increased mortality in patients with persistent VRE BSIs and the limited number of treatment options available, further insight into risk factors associated with persistence is warranted. We aimed to identify risk factors associated with persistent VRE BSIs, including those related to patient management, such as antimicrobial selection and source control.

## Methods

### Study design and population

We conducted a single center, retrospective, case-control study. Adult patients (≥ 18 years old) admitted to Stanford Hospital (Stanford, California, USA) between 2016 and 2020 with ≥ 1 blood culture positive for vancomycin resistant *Enterococcus *spp. were screened for inclusion. Case patients included those who had an episode of persistent bacteremia, defined as bacteremia for ≥ 4 days despite VRE-directed therapy. Control patients had non-persistent bacteremia, defined as documented clearance of blood cultures within 4 days of active treatment initiation. Patients were excluded if they had incomplete data records, did not have repeat blood cultures drawn after the initial index culture, did not receive VRE-directed therapy, or expired within 4 days of treatment initiation. This study was reviewed and deemed to be non-human subjects research by the Stanford University School of Medicine Panel on Human Subjects in Medical Research.

### Data collection

Patient data was extracted from the medical record by a single trained reviewer, using a structured data collection form within REDCap (Research Electronic Data Capture, Stanford University) [17, 18]. Patient demographics, including comorbidities, presence or absence of a central line or prosthetic device at the time of index blood culture, infection site, antimicrobial management and outcomes were collected. The Charlson comorbidity index (CCI) was used to provide a composite score of comorbid conditions. The severity of bacteremia at the time of the initial positive VRE blood culture was assessed using the Pitt bacteremia score, with most significant values within 48 h of index culture.

### Definitions

Duration of bacteremia was defined as the number of days between the first positive and last positive blood culture. Recurrent VRE BSI was defined as a new positive blood culture result for VRE following at least 14 days of negative blood cultures after receiving active therapy. Breakthrough VRE BSI was defined as initial clearance of bacteremia followed by a subsequent positive blood culture within 14 days of the initial index culture. Severe neutropenia was defined as an absolute neutrophil count < 500 cells/mm^3^. Source of VRE bacteremia and necessity for source control was determined via review of treating physicians’ notes and the available clinical and diagnostic data, including vital signs, laboratory and microbiology parameters, and imaging. Patients with identified sources of infection were further evaluated to determine if a source control intervention was required. Source was considered uncontrolled if an intervention was necessary and not performed.

### Microbiologic data

Standard blood culture collection policy was to obtain two sets of blood cultures: one bottle of aerobic and one bottle of anaerobic culture medium in set one, and two bottles of aerobic culture medium in set two. The first positive blood isolate from each patient was used for microbiologic and molecular assessments. Screening for vancomycin resistance was performed by polymerase chain reaction detection of the *vanA* gene. Antimicrobial susceptibilities were subsequently determined using an automated system (MicroScan WalkAway Plus System (Beckman Coulter, Brea, CA)) and interpreted according to Clinical and Laboratory Standards Institute (CLSI) guidance at the time of the index blood culture. Daptomycin minimum inhibitory concentrations (MICs) of ≥ 4 mg/L were confirmed via E test.

### Outcomes

The primary outcome was risk factors associated with persistent VRE BSI. Secondary outcomes included in-hospital mortality and rates of breakthrough and recurrent bacteremia.

### Statistical analysis

Clinical characteristics were compared using independent t tests for continuous data and chi square tests for discrete data. A logistic regression model was constructed to evaluate the association between specific factors for persistent bacteremia. Risk factors chosen for inclusion in the multivariable logistic regression model were determined by plausibility of clinical significance as perceived by the researchers. Risk factors included in the regression model were age, gender, CCI, cirrhosis, history of malignancy (hematologic and solid tumor), solid organ transplant history, severe neutropenia, Pitt bacteremia score, ICU stay at index culture, 4 out of 4 positive blood cultures, lack of source control, and receipt of an oxazolidinone within the initial 72 h of treatment. Results were presented as odds ratios (OR) with corresponding 95% confidence intervals (CI). Model fit was assessed using the Hosmer-Lemeshow goodness-of-fit test, which evaluated the null hypothesis that the observed event rates were no different from the expected event rates [19]. Subgroup analyses were performed among patients who received daptomycin within the initial 72 h of treatment and source control interventions. Statistical significance was set at a two-sided alpha < 0.05. All statistical analyses were performed using Stata SE version 15 (Stata Corp., College Station, TX).

## Results

### Study population

**Fig. 1:**
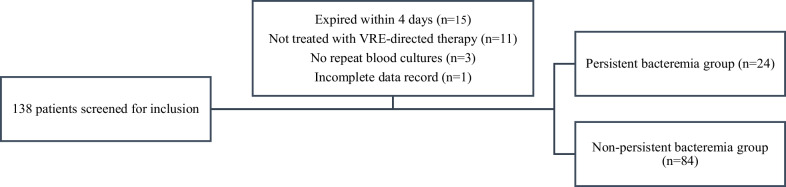
Study population

During the study period, 138 patients had a blood culture positive for VRE. A total of 30 patients were excluded. The most common reason for exclusion was death within four days of VRE index culture (Fig. 1). Of the patients that met inclusion criteria (n = 108), 24 (22.2%) had persistent and 84 (77.8%) had non-persistent VRE bacteremia. The median (IQR) duration of bacteremia was 7 (6–11) days versus 1 (1–2) day in the persistent and non-persistent group, respectively.

### Primary outcome

No significant difference in baseline demographics were identified except for a higher number of patients on intermittent hemodialysis in the persistent group (33.3% versus 14.3%; p = 0.034; Table 1). All patients in the persistent group and 96% (81/84) in the non-persistent group had blood cultures positive with *Enterococcus faecium*. More patients in the persistent group had 4 out of 4 blood culture bottles positive (54.2% versus 21.4%; p = 0.002), and more patients in the non-persistent group had 1 out of 4 bottles positive (31.0% versus 8.3%; p = 0.026; Table 1).

**Table 1:** Characteristics of patients with persistent and non-persistent VRE Bacteremia

Characteristic	Persistent Bacteremia (N = 24)	Non-persistent Bacteremia (N = 84)	P value
Age (yr), median (IQR)	53 (22.5)	60 (21)	0.0467
Male	14 (58.3%)	54 (64.3%)	0.594
Prior antibiotic exposure			
Vancomycin (IV)	10 (41.7%)	35 (41.7%)	1
Vancomycin (PO)	0 (0.0%)	2 (2.4%)	0.445
Cephalosporins	11 (45.8%)	32 (38.1%)	0.495
Carbapenems	5 (20.8%)	22 (26.2%)	0.593
Fluoroquinolones	15 (62.5%)	48 (57.1%)	0.639
Metronidazole	6 (25.0%)	19 (22.6%)	0.807
Prior VRE infection	1 (4.2%)	8 (9.5%)	0.402
Underlying condition			
Solid tumor	2 (8.3%)	15 (17.9%)	0.259
Hematologic malignancy	5 (20.8%)	17 (20.2%)	0.949
History of BMT	0 (0.0%)	6 (7.1%)	0.178
History of SOT	10 (41.7%)	27 (32.1%)	0.386
Renal failure	9 (37.5%)	31 (36.9%)	0.958
Liver cirrhosis	8 (33.3%)	18 (21.4%)	0.229
Diabetes mellitus	6 (25.0%)	32 (38.1%)	0.236
Recent GI surgery	5 (20.8%)	22 (26.2%)	0.593
Grade 3–4 mucositis	0 (0.0%)	6 (7.1%)	0.178
Severe neutropenia	2 (8.3%)	6 (7.1%)	0.844
ECMO	2 (8.3%)	17 (20.2%)	0.177
CRRT	8 (33.3%)	28 (33.3%)	1
Intermittent hemodialysis	8 (33.3%)	12 (14.3%)	0.034
Charlson Comorbidity Index, median (IQR)	5 (4–7)	5 (3–7)	0.704
Pitt bacteremia score, median (IQR)	2.5 (1–8)	2 (0–6)	0.213
ICU stay during admission	14 (58.3%)	51 (60.7%)	0.834
ICU stay at index culture	11 (45.8%)	39 (46.4%)	0.959
Source of infection			
Intra-abdominal	12 (50.0%)	44 (52.4%)	0.386
Undifferentiated	5 (20.8%)	22 (26.2%)	
Central line	1 (4.2%)	8 (9.5%)	
Other (skin, urinary, pleural fluid, device-related)	6 (25.0%)	10 (11.9%)	
Central line present at index culture	16 (66.7%)	57 (67.9%)	0.912
Central line removed	12 /16 (75%)	41 / 57 (71.9%)	0.808
*E. faecalis* spp. BSI	0 (0.0%)	3 (3.6%)	0.348
*E. faecium* spp. BSI	24 (100.0%)	81 (96.4%)	0.445
Polymicrobial bacteremia	3 (12.5%)	16 (10.1%)	0.458
Concurrent candidemia	1 (4.2%)	6 (7.1%)	0.601
1 of 4 positive index blood culture bottles	2 (8.3%)	26 (31.0%)	0.026
4 of 4 positive index blood culture bottles	13 (54.2%)	18 (21.4%)	0.002
Treatment (initial 72 h)			
Daptomycin	15 (62.5%)	47 (56.0%)	0.567
Linezolid	13 (54.2%)	42 (50.0%)	0.719
Tedizolid	3 (12.5%)	10 (11.9%)	0.937
Tigecycline	0 (0.0%)	1 (1.2%)	0.591
Omadacycline	0 (0.0%)	1 (1.2%)	0.591
ID consult	22 (91.7%)	82 (97.6%)	0.173

The only significant risk factor for persistence pertaining to management identified in the bivariate analysis was an uncontrolled source of infection. The most common source of infection in both groups was intra-abdominal, accounting for 50% (12/24) and 52.4% (44/84) of patients in the persistent and non-persistent groups, respectively. Other identified sources of infection included central line-related (4.2% [1/24] in the persistent group and 9.5% [8/84] in the non-persistent group), undifferentiated (20.8% [5/24] in the persistent group and 26.2% [22/84] in the non-persistent group), and other (25% [6/24] in the persistent group and 11.9% [10/84] in the non-persistent group). Of those in the persistent group, 67% (16/24) of patients required a source control intervention versus 39% (33/84) of patients in the non-persistent VRE BSI group. In the subgroup analysis of those patients, 56% (9/16) underwent a source control intervention in the persistent group versus 91% (30/33) in the non-persistent group. Additionally, the median (IQR) time to source control intervention was 9 (2.5–17.5) days in the persistent group and 3 (1–4) days in the non-persistent group (p = 0.0127; Additional file 1: Table S1). No differences were observed between patients initially treated with daptomycin, oxazolidinones, or tetracyclines. Additionally, in the subgroup analysis of patients treated with daptomycin (n = 62), no differences were observed based on daptomycin MIC or the initial daptomycin weight-based dose utilized (Additional file 1: Table S2).

**Fig. 2:**
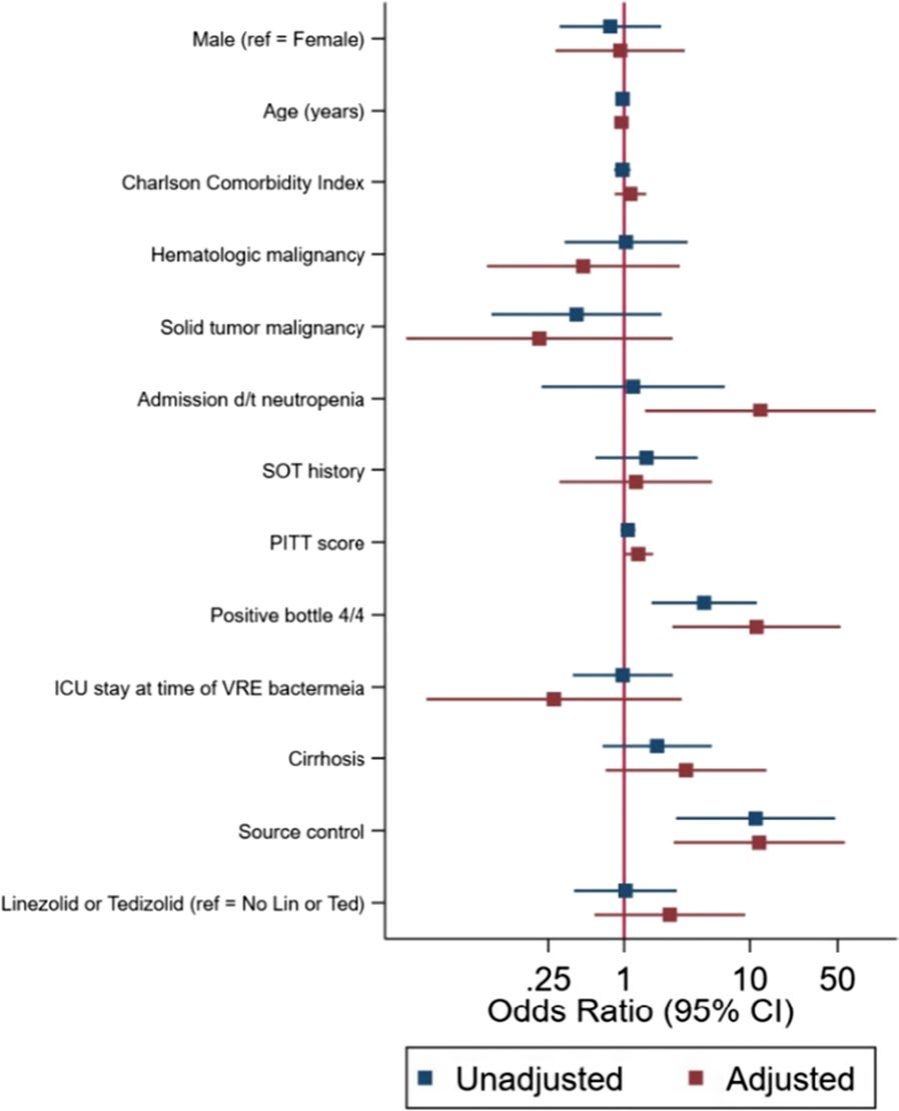
Log odds of regression parameters

In the logistic regression analysis (Fig. 2), patients with severe neutropenia at initial index culture had a 12.12 higher odds of having persistent bacteremia compared to patients without severe neutropenia (95% CI 1.47, 100.26) while controlling for the other covariates. Four out of 4 positive index blood culture bottles were associated with an 11.29 higher odds of having persistent bacteremia (95% CI 2.42, 52.68). Lack of source control was associated with an 11.88 higher odds of having persistent bacteremia compared to those who obtained source control or had an infection that did not require a source control intervention (95% CI 2.48, 56.91). The Hosmer-Lemeshow goodness-of-fit test p-value was 0.13, indicating that in our model the observed event rates did not deviate from the expected event rates, thus the the regression model fit the data well.

### Secondary outcomes

Table 2 summarizes the results of the unadjusted secondary outcome analysis. In-hospital mortality was observed in 58% versus 40% of patients in the persistent and non-persistent groups (p = 0.121). There were no statistically significant differences in recurrent bacteremia (17% versus 6%; p = 0.090) or breakthrough bacteremia (13% versus 7%; p = 0.402) between groups.

**Table 2:** Clinical outcomes of patients with persistent and non-persistent VRE Bacteremia

Outcome	Persistent Bacteremia (N = 24)	Non-persistent Bacteremia (N = 84)	P value
In-hospital mortality	14 (58%)	34 (40%)	0.121
Recurrent bacteremia	4 (17%)	5 (6%)	0.090
Breakthrough bacteremia	3 (13%)	6 (7%)	0.402

## Discussion

Prior studies that have identified risk factors for persistent BSIs have almost exclusively focused on *Staphylococcus aureus*. However, persistent bacteremia due to VRE is also often encountered in clinical practice. In our cohort, 22% of patients experienced persistent bacteremia for four days or more despite VRE-directed therapy, an incidence almost identical to that reported in a recent prospective multicenter study [10]. While other publications have shown that persistent VRE bacteremia is an independent predictor of mortality, none have sought to elucidate risk factors for persistence [7–11]. As such, our findings fill a much-needed gap in identifying those at high risk of persistent VRE BSI who may benefit from more aggressive management strategies.

Delayed source control has been well-described as a risk factor for persistent *Staphylococcus aureus* bacteremia [12]. Our findings indicated similar results for VRE, as patients in the persistent group were more likely to have an infection source that required a source control intervention, and the corresponding median time to intervention was also longer. This coincides with the breakdown of primary infection source between groups. The non-persistent group had a higher incidence of unknown infection sources. In the absence of an obvious source, many of these were presumably secondary to gastrointestinal translocation, where source control interventions may not be indicated.

Severe neutropenia was not a statistically significant risk factor in the unadjusted analysis, but it was significant in the adjusted analysis when controlling for confounders. To our knowledge, neutropenia has not been previously described as a risk factor for persistent *Staphylococcus aureus* bacteremia. However, it has been identified as a strong risk factor for VRE colonization and infection [1, 4]. Additionally, in a retrospective cohort analysis, Bhavnani et al. similarly found that patients with multiple positive blood cultures for *E. faecalis* or *E. faecium* had higher proportions of hematologic malignant neoplasms or neutropenia [20]. Our findings expand upon these results and support the assertion that neutropenia should be considered an important risk factor for persistent VRE bacteremia.

To our knowledge, 4 out of 4 positive index blood cultures has never been identified as a risk factor for persistent bacteremia. We collected number of positive blood culture bottles as a surrogate for overall bacterial burden. While acknowledging this is not a perfect marker, it can be easily applied in real-world clinical practice. In our study, 31.0% of patients in the non-persistent VRE BSI group had a single positive blood culture versus just 8.3% in the persistent group. Some clinicians may assert that a single positive blood culture for VRE may be more consistent with contamination rather than infection. Sexton et al. previously suggested that the National Healthcare Safety Network (NHSN) definition of primary Enterococcal BSI should require at least 2 positive blood cultures for this reason [21, 22]. While the significance of a single positive blood culture with Enterococcus may warrant additional study, our results do indicate that 4 out of 4 positive index blood cultures is associated with a higher risk of persistent VRE bacteremia.

Another interesting finding was the lack of therapy implications on persistent VRE bacteremia. Within the subgroup of patients that received linezolid or tedizolid for management within the first 72 h, there was no difference in persistent bacteremia, suggesting that initial treatment with a “bacteriostatic” antimicrobial is not a risk factor for persistence. Studies comparing treatment with linezolid to daptomycin for VRE bacteremia have yielded conflicting results, so these findings were not unexpected [13–15]. In contrast, one management recommendation well-supported by existing literature, prompting a CLSI breakpoint revision in 2019, is that daptomycin doses of ≥ 8 mg/kg/day are required for optimal treatment of VRE (particularly when the MIC is 3–4 mg/L) [13]. Surprisingly, in our subgroup analysis of patients treated with daptomycin, there was not a significant difference in initial dosages or daptomycin MIC between the persistent and non-persistent groups. This may be explained by low numbers of patients in the daptomycin subgroup analysis (particularly in the persistent VRE BSI group).

We observed a numerically higher incidence of mortality, recurrent bacteremia, and breakthrough bacteremia in the persistent VRE group, but this did not meet statistical significance. That said, the 2009 Infectious Disease Society of America guideline for intravascular catheter-related infections state that Enterococcal bacteremia persisting > 4 days is associated with mortality. [9]. Additionally, multiple prior studies have found that persistent Enterococcal BSI is an independent predictor of poor outcome. This was most recently confirmed in the study by Contreras et al., in which microbiologic failure (at ≥ 4 days from index culture) had a HR of 5.03 for in-hospital mortality [7–11]. The lack of a statistically significant difference in mortality between groups observed in our study may be due to Type II error owing to limitations of our sample size.

This study has several limitations. First, this was a retrospective single-center study with a relatively small sample size and therefore may not be generalizable to all patient populations. Only a limited number of variables could be included in the multivariable analysis to avoid over-fitting. Because of this, only variables considered to have the highest plausibility of clinical significance were included. Second, 25% of VRE BSI cases had an unknown source, as it was difficult in some cases to determine retrospectively. This is a common limitation given the frequency of VRE BSI cases secondary to gastrointestinal translocation, and other retrospective Enterococcal BSI studies have reported a similar incidence of ~ 10–36% of patients with VRE BSI secondary to unknown source [5, 8]. Third, blood culture practices were heterogeneous throughout the study period. Repeat blood cultures were not always drawn at the same intervals, which could affect the duration of bacteremia reported. Additionally, some patients only had one set of blood cultures drawn, and not all institutions may utilize our same blood culture collection approach, in which one set may constitute two aerobic bottles or one anaerobic and one aerobic bottle. Fourth, while we evaluated the number of positive blood culture bottles, we did not capture the time from specimen collection to blood culture positivity, which has been associated with severity of infection. Future studies should consider evaluating time to positivity as a marker for persistent VRE bacteremia. Finally, “persistent” VRE BSI has not been formally defined, and the definition used in this study may be controversial amongst practitioners. This, however, would not be unique to VRE, as the definition of persistent *Staphylococcus aureus* BSI has been heavily debated and studied extensively, and still remains variable in the literature ranging anywhere from 2 to 7 days [23]. Nonetheless, the definition used in our study was supported by the mortality impact of ≥ 4 days of persistent VRE BSI noted in existing studies.

## Conclusion

Our results indicated that 22% of patients with VRE BSI, particularly those with severe neutropenia, 4 out of 4 positive index blood culture bottles, and an uncontrolled source had persistent bacteremia despite 4 days of appropriate antibiotic treatment. We noted a numerically higher incidence of mortality, recurrent bacteremia, and breakthrough bacteremia in the persistent VRE BSI group. Larger prospective studies should be conducted to confirm the mortality impact and explore management strategies for persistent VRE BSI. While awaiting further data, clinicians may consider aggressive VRE BSI management, such as early source control interventions, for neutropenic patients or those with 4 out of 4 positive index blood culture bottles who are at risk for persistent VRE bacteremia.

## Supplementary Information

**Additional file 1: Table S1.** Source Control in Patients with Persistent and Non-Persistent VRE Bacteremia. **Table S2.** Daptomycin Utilization in Patients with Persistent and Non-Persistent VRE Bacteremia

## Data Availability

The datasets used and/or analyzed during the current study are available from the corresponding author on reasonable request.

## Abbreviations

VRE: Vancomycin resistant Enterococci
BSI: Blood stream infections
CCI: Charlson Comorbidity Index
CLSI: Clinical and Laboratory Standards Institute
MIC: Minimum inhibitory concentration
ICU: Intensive care unit
OR: Odds ratio
CI: Confidence interval
IQR: Interquartile range

## References

[1] Vydra J (2012). Enterococcal bacteremia is associated with increased risk of mortality in recipients of allogeneic hematopoietic stem cell transplantation. Clin Infect Dis.

[2] Noskin GA, Peterson LR, Warren JR (1995). *Enterococcus faecium* and *Enterococcus faecalis* Bacteremia: acquisition and outcome. Clin Infect Dis.

[3] Thomas P, Peggy L, Vincent HT, Rybak MJ (2002). Clinical outcomes for patients with bacteremia caused by vancomycin-resistant enterococcus in a level 1 trauma center. Clin Infect Dis.

[4] Billington EO, Incidence (2014). Risk factors, and outcomes for *Enterococcus* spp. blood stream infections: a population-based study. Int J Infect Dis.

[5] Zasowski EJ, Claeys KC, Lagnf AM, Davis SL, Rybak MJ (2016). Time is of the essence: the impact of delayed antibiotic therapy on patient outcomes in hospital-onset enterococcal bloodstream infections. Clin Infect Dis.

[6] Ye J-J (2018). Clinical characteristics and treatment outcomes of vancomycin-resistant *Enterococcus faecium* bacteremia. J Microbiol Immunol Infect.

[7] DiazGranados CA, Zimmer SM, Klein M, Jernigan JA (2005). Comparison of mortality associated with vancomycin-resistant and vancomycin-susceptible enterococcal bloodstream infections: a meta-analysis. Clin Infect Dis.

[8] Contreras GA (2021). Contemporary clinical and molecular epidemiology of vancomycin-resistant enterococcal bacteremia: a prospective multicenter cohort study (VENOUS I). Open Forum Infect Dis..

[9] Mermel LA (2009). Clinical practice guidelines for the diagnosis and management of intravascular catheter-related infection: 2009 update by the Infectious Diseases Society of America. Clin Infect Dis.

[10] Chuang Y-C (2018). A retrospective clinical comparison of daptomycin vs daptomycin and a beta-lactam antibiotic for treating vancomycin-resistant *Enterococcus faecium* bloodstream infections. Sci Rep.

[11] Bhavnani SM (2000). A nationwide, multicenter, case-control study comparing risk factors, treatment, and outcome for vancomycin-resistant and -susceptible enterococcal bacteremia. Diagn Microbiol Infect Dis.

[12] Chong YP (2013). Persistent *Staphylococcus aureus* bacteremia: a prospective analysis of risk factors, outcomes, and microbiologic and genotypic characteristics of isolates. Med (Baltim).

[13] Kanjilal S, Kalil AC, Klompas M (2018). What is the best treatment for vancomycin-resistant enterococcal bloodstream infections?*. Crit Care Med.

[14] Patel R, Gallagher JC (2015). Vancomycin-resistant enterococcal bacteremia pharmacotherapy. Ann Pharmacother.

[15] Barber KE, King ST, Stover KR, Pogue JM (2015). Therapeutic options for vancomycin-resistant enterococcal bacteremia. Expert Rev Anti-infective Therapy.

[16] Miller WR, Murray BE, Rice LB, Arias CA (2016). Vancomycin-resistant enterococci: therapeutic challenges in the 21st century. Infect Dis Clin North Am.

[17] Harris PA (2019). The REDCap consortium: building an international community of software platform partners. J Biomed Inform.

[18] Harris PA (2009). Research electronic data capture (REDCap)—a metadata-driven methodology and workflow process for providing translational research informatics support. J Biomed Inform.

[19] Hosmer DW, Lemesbow S (1980). Goodness of fit tests for the multiple logistic regression model. Commun Stat Theory Methods.

[20] Bhavnani SM (2000). A nationwide, multicenter, case-control study comparing risk factors, treatment, and outcome for vancomycin-resistant and -susceptible enterococcal bacteremia☆. Diagn Microbiol Infect Dis.

[21] Freeman JT, Chen LF, Sexton DJ, Anderson DJ (2011). Blood culture contamination with Enterococci and skin organisms: implications for surveillance definitions of primary bloodstream infections. Am J Infect Control.

[22] Sexton DJ, Chen LF, Anderson DJ (2010). Current definitions of central line–associated bloodstream infection is the emperor wearing clothes?. Infect Control Hosp Epidemiol.

[23] Kuehl R (2020). Defining persistent *Staphylococcus aureus* bacteraemia: secondary analysis of a prospective cohort study. Lancet Infect Dis.

