# Supplementary material for: Risk factors and outcomes associated with persistent vancomycin resistant Enterococcal Bacteremia
Source: BMC Infect Dis. 2022 Nov 16;22:855. doi: 10.1186/s12879-022-07864-8 (PMC9670444; doi:10.1186/s12879-022-07864-8)
Supplement: Supplementary file 1 — Additional file 1: Table S1. Source Control in Patients with Persistent and Non-Persistent VRE Bacteremia. Table S2. Daptomycin Utilization in Patients with Persistent and Non-Persistent VRE Bacteremia [file 12879_2022_7864_MOESM1_ESM.docx]

**Additional file**

Table S1. Source Control in Patients with Persistent and Non-Persistent VRE Bacteremia

| Characteristic | Persistent Bacteremia  (N=24) | Non-persistent Bacteremia (N=84) | P value |
| --- | --- | --- | --- |
| Source control intervention | | | |
| Yes/Not required | 17 (70.8%) | 81 (96.4%) | <0.001 |
| No | 7 (29.2%) | 3 (3.6%) |  |
| Intervention within 72 hours of  index culture | | | |
| Yes | 2 / 9 (22.2%) | 21 / 29 (72.4%) | 0.007 |
| No | 7 / 9 (77.8%) | 8 / 29 (27.6%) |  |
| Median time to source control (d) | 9 (2.5-17.5) | 3 (1-4) | 0.0127 |

Table S2. Daptomycin Utilization in Patients with Persistent and Non-Persistent VRE Bacteremia

| Characteristic | Persistent Bacteremia  (N=24) | Non-persistent Bacteremia (N=84) | P value |
| --- | --- | --- | --- |
| Daptomycin MIC | | | |
| 4 or greater mg/L | 15 (62.5%) | 57 (67.9%) | 0.454 |
| 2 mg/L | 8 (33.3%) | 19 (22.6%) |  |
| Less than 2 mg/L | 1 (4.2%) | 8 (9.5%) |  |
| Initial daptomycin dose | | | |
| >10 mg/kg | 2 (8.3%) | 7 (8.3%) | 0.736 |
| 8-9.9 mg/kg | 4 (16.7%) | 19 (22.7%) |  |
| <8 mg/kg | 9 (37.5%) | 22 (26.2%) |  |
| N/A | 9 (37.5%) | 36 (42.9%) |  |
